# In the Era of Molecular Pathology, the Role of Morphological Changes in Megakaryocytes in Bone Marrow Aspiration in Cases of Isolated Thrombocytopenia

**DOI:** 10.7759/cureus.74336

**Published:** 2024-11-24

**Authors:** Deepshikha Verma, Lal Pranay Singh, Preeti Mandloi, Rajnikant Ahirwar

**Affiliations:** 1 Department of Pathology, Atal Bihari Vajpayee Government Medical College, Vidisha, IND

**Keywords:** bone marrow aspiration, immune thrombocytopenia purpura, megakaryocytes, morphological changes, thrombocytopenia

## Abstract

Background: The most typical cause of thrombocytopenia is immune-mediated thrombocytopenic purpura (ITP). Thrombocytopenia can cause insufficient clot formation and increase the risk of bleeding. Bone marrow aspiration is commonly used for this purpose. ITP was primarily caused by immune-mediated platelet destruction and a megakaryocyte maturation abnormality. The objectives of this study were to evaluate the inter-observer reliability of bone marrow examinations and to identify different megakaryocyte morphological characteristics observed in ITP bone marrow aspiration smears.

Materials and methods: A prospective study was done on 100 cases of bone marrow aspirations of thrombocytopenia in 1 year from 2019 to 2020 in Central India’s tertiary care center. Reporting was done by hematopathologists with particular emphasis on megakaryocyte morphology.

Results: A total of 100 cases of bone marrow aspiration were done in one year of thrombocytopenia, out of which 35 (35%) cases were of primary immune thrombocytopenia in which morphological alterations were noted, which constitutes the second most common cause of bone marrow aspiration. In contrast, acute leukemia was the first leading cause. As long as a thorough history and physical examination are conducted, a complete blood count, a peripheral blood smear, and routine coagulation studies reveal no abnormalities other than thrombocytopenia, the literature suggests that performing a routine bone marrow examination to diagnose ITP is unnecessary. Although ITP bone marrow smears showed morphological changes in megakaryocytes, they can be significant as well as insignificant in view of classifying it as myelodysplastic syndrome (MDS).

Conclusion: This study indicates significant abnormalities in the megakaryocytes of ITP patients. Understanding the morphological changes of megakaryocytes in bone marrow aspirates is equally important in the era of molecular diagnostics. It can enhance the accuracy of diagnosis for a variety of hematological disorders and enable the implementation of suitable treatment measures.

## Introduction

According to William Harvey, "the fountain of life and principal seat of the soul" is blood. The marrow of bones is where our blood is sown [1]. To comprehend and treat various hematological disorders, bone marrow analysis is essential. Pancytopenia is characterized by the simultaneous occurrence of anemia, leukopenia, and thrombocytopenia. According to reports, the annual incidence of immune-mediated thrombocytopenic purpura (ITP) ranges from 1 to 5 per 100,000 adults, and because of its propensity for chronicity, prevalence exceeds incidence [2,3]. The age distribution of adult patients with ITP shows a significant decline after childhood, which tends to increase after 60 years. Despite the general gender inclination towards females, epidemiological studies have shown that the incidence of ITP has increased after 70 years, with a preponderance of males [2,3].

Megakaryocytes are uncommon myeloid cells that mostly reside in the bone marrow and comprise less than 1% of the population [1]. A single megakaryocyte can release up to thousands of platelets as a result of a series of remodeling processes that megakaryocytes use to create platelets. Each megakaryocyte is thought to be capable of producing 1000-5000 platelets [1].

One of the most prevalent forms of megakaryocytic thrombocytopenia, ITP, is characterized by hyperplasia of megakaryocytes in bone marrow [4]. Numerous studies have shown that in these situations, megakaryocytes mature more quickly [5]. However, a diagnosis of ITP is always considered a diagnosis of exclusion. It can appear as a primary condition or secondary condition in conjunction with other diseases like autoimmune conditions and infectious causes. The diagnosis uses clinical history and a peripheral smear that is within normal limits except for thrombocytopenia. However, bone marrow examination is frequently done to rule out leukemia, myelodysplastic disease, or aplastic anemia. However, in cases of isolated thrombocytopenia, a bone marrow examination is not advised by consensus standards [6].

## Materials and methods

From 2019 to 2020, a prospective study was conducted on patients with thrombocytopenia who underwent bone marrow aspiration (BMA) under the IRB institution of GMC Bhopal. Patients with bleeding manifestations (not due to thrombocytopenia), in which BMA is contraindicated, and non-cooperative patients were excluded from this study. All the cases in this study of thrombocytopenia diagnosed on an automated hematology analyzer had low platelet counts of less than 1,50,000/cumm, confirmed by peripheral smear, and divided into the mild, moderate, and severe categories. The mild category was generally only considered for BMA clinically indicated. The consent form, along with all the required clinical data, was documented. Bone marrow aspiration was performed in those cases following aseptic conditions. After that, the BMA smears were stained with Leishman stain.

BMA smears were reported using the recommended protocol, and results were recorded. Megakaryocyte counts were noted as counts per 10 low power fields (LPFs), and then they were divided into four groups [4], as shown in Table 1.

**Table 1:** Megakaryocyte reporting according to count LPF: Low power field

Reporting of megakaryocytes	Megakaryocytic Count
Absent	No megakaryocyte seen
Decreased	1 per 5-10 LPF
Normal	1 per 1-3 LPF
Increased	>2 per LPF

Both dysplastic (multiple separated nuclei, micro-megakaryocytes, and hypogranular form) and non-dysplastic (emperipolesis, bare naked nuclei, immature, cytoplasmic vacuolization, and budding) megakaryocytic abnormalities were looked for in at least 30 megakaryocytes. Dysplastic alterations were only considered substantial when the observed anomalies occurred in 10% or more megakaryocytes.

## Results

Out of 100 cases examined for thrombocytopenia, 35 (35%) cases were diagnosed as ITP. Patients with ITP mainly presented as bleeding manifestations. However, severe bleeding manifestations were uncommon in the present study. Platelet count in patients presented as thrombocytopenia is classified according to the count described in Table 2.

**Table 2:** Platelet count in patients presented with thrombocytopenia

Platelet count	No. of Patients (%)
<20,000/cumm	6 (17.14%)
20,000-50,000/cumm	15 (42.8%)
50,000-1 lac/cumm	10 (28.57%)
1 lac-1.5lac/cumm	4 (11.4%)
Total	35

**Figure 1:**
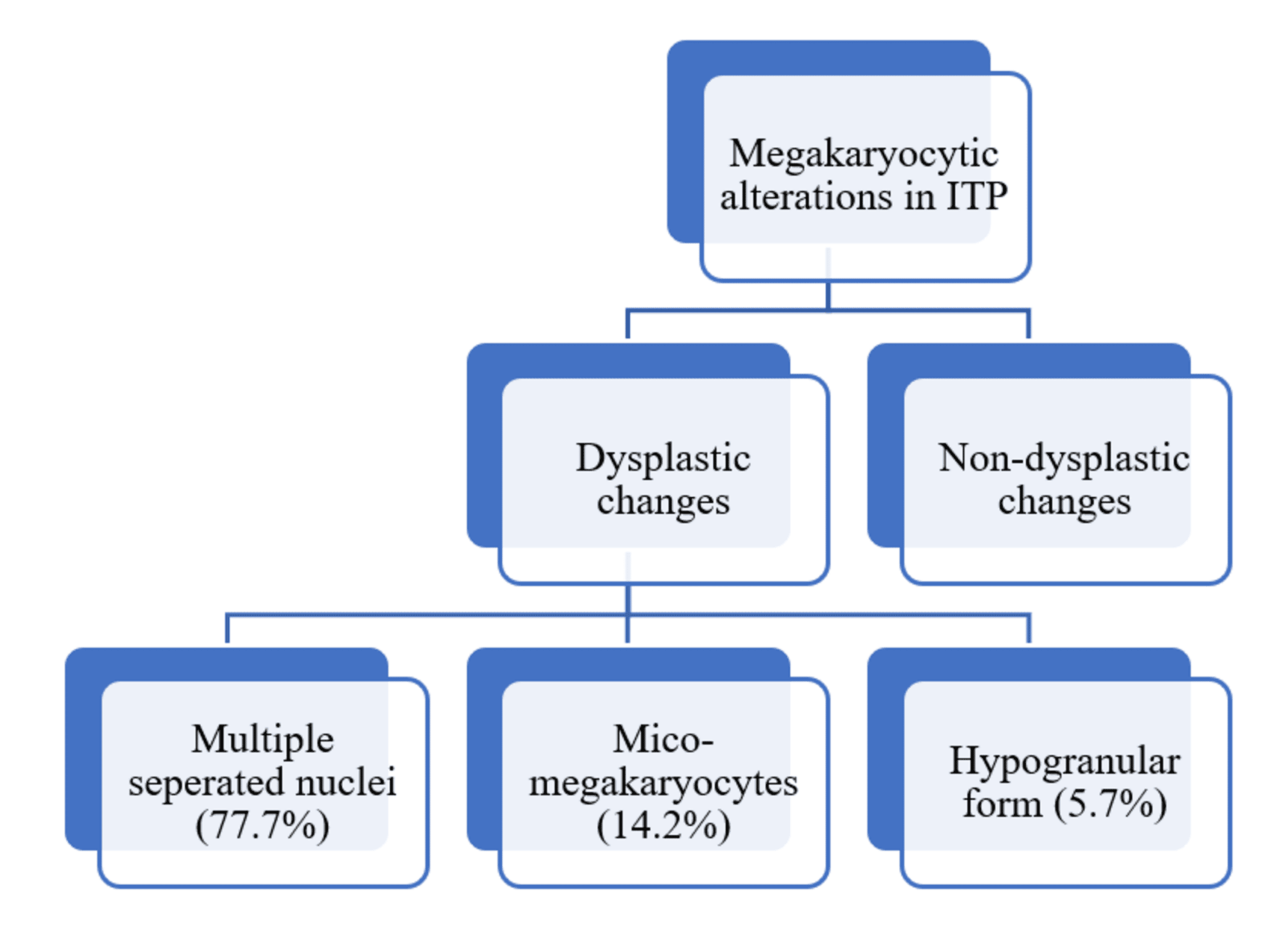
Dysplastic changes in megakaryocytes in ITP patients Megakaryocyte alteration seen in the patients with immune-mediated thrombocytopenic purpura (ITP)

Platelet count encountered in different patients of ITP with a mean platelet count of 54,971/cumm. 68.57% of cases were normocellular in bone marrow aspiration. In the present study, all cases of ITP (100%) show increased megakaryocytes in bone marrow aspiration smears, which is a usual finding. Also, dysplastic features were observed in the morphology of megakaryocytes in ITP. Multiple separated nuclei were commonly observed (77.77%), followed by micro-megakaryocytes (14.7%). Among non-dysplastic changes, emperipolesis was observed in all cases of ITP, followed by an immature form. Morphological alteration (dysplastic changes) in megakaryocytes in ITP patients is seen in Figure 1.

**Figure 2:**
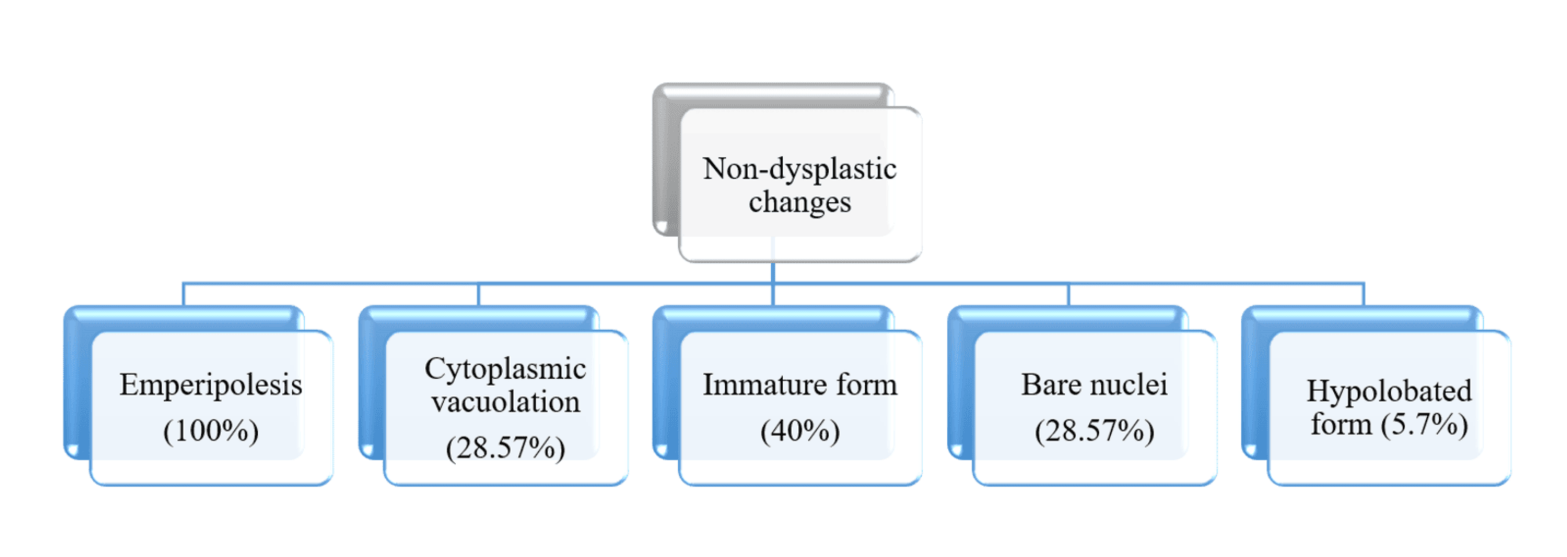
Non-dysplastic changes in megakaryocytes in ITP patients

Non-dysplastic changes observed in megakaryocytes in bone marrow aspiration smears were graphically presented in Figure 2.

**Figure 3:**
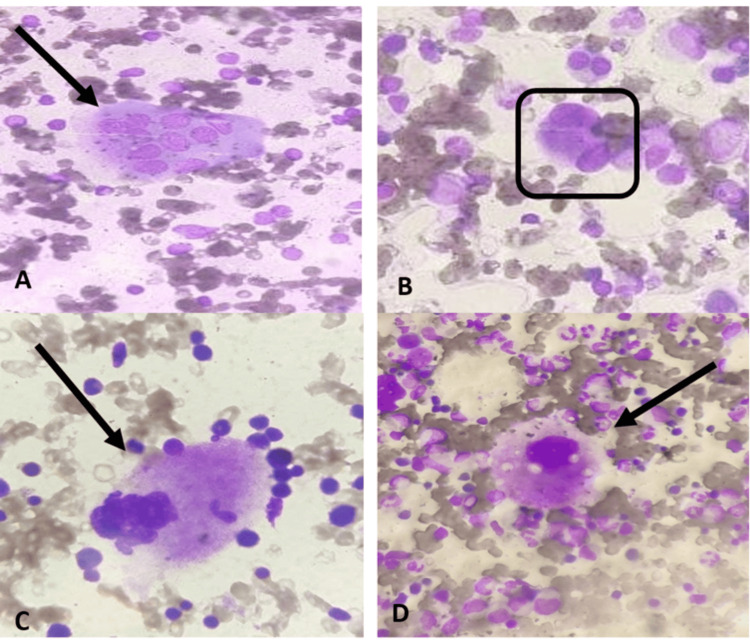
Morphological changes observed in megakaryocytes in bone marrow aspirate smear. Figure 3A shows multiple separated nuclei of the megakaryocytes highlighted by the black arrow. Figure 3B shows micro-megakaryocytes inside the black square. Figure 3C shows emperipolesis in the megakaryocyte (with a black arrow showing engulfing of neutrophil inside the cytoplasm of megakaryocytes). Figure 3D is showing cytoplasmic vacuolization in megakaryocytes pointed by a black arrow.

The presence of abnormal megakaryocytes, which included multiple separated nuclei (Figure 3A), micro-megakaryocytes (Figure 3B), and hypogranular forms (dysplastic changes), was considered as dysmegakaryocytopoiesis while non-dysplastic changes like emperipolesis (Figure 3C) and cytoplasmic vacuolation (Figure 3D) were seen in Figure 3.

Studies considered that these dysplastic changes were present only in Myelodysplastic Neoplasm (MDN), previously termed Myelodysplastic Syndrome (MDS) condition, but various studies proved that these changes were not only shown by MDS condition but also other hematological disorders too [7-9]. Our study also favors the same, and most of these changes were shown by ITP in our study.

## Discussion

In the present study, increased megakaryocytes were observed in all the cases of ITP, which is 100%, which is quite similar to the analysis done by Muhury et al. [7] (94.9% cases), Bhasin et al. [8] (100% cases), Gupta et al. [9] (82.3%), Choudhary PK et al. [10] (91%), Sandeep et al. [11] (100%), and Vinayakmurthy et al. [12] (80%). Marrow megakaryocytes may have been driven to produce more rapidly due to immune-mediated platelet destruction in the spleen and other reticuloendothelial organs [9].

Dysplastic megakaryocytes were defined as megakaryocytes with numerous separated nuclei. Micro-megakaryocytes are cells with a single or bilobed nucleus smaller than large lymphocytes or monocytes. It was believed that if cytoplasmic processes started emerging from their surfaces, they displayed platelet budding. Hypogranular types were megakaryocytes with pale cytoplasm and few or no granules. The type of cell included in megakaryocytes during emperipolesis was also noted. Similar to Murphy et al., ITP most frequently displayed numerous separated nuclei in dysplastic alterations [7]. According to Choudhary et al. [10], the most frequent cause of multiple separated nuclei is megaloblastic anemia, which they attribute to reduced and inadequate Deoxyribonucleic Acid (DNA) synthesis that results in a nuclear maturation deficiency.

One well-known essential feature of myelodysplastic syndrome is dys-megakaryocytopoiesis, which can manifest as micro-megakaryocytes, multiple separated nuclei, or hypogranular forms. In certain studies, this is an independent prognostic factor. The present study found that the hypo-granular shape was the least apparent dysplastic feature (5.7%). According to various studies, megakaryocytic hypogranulation is the most diagnostic feature of myelodysplasia in Myelodysplastic Syndrome (MDS). Similar to Vinayakmurthy et al. [12], ITP was the most frequent cause of micro-megakaryocytes (44.45% among all cases of thrombocytopenia in the one-year study period). In contrast, Muhury et al. [7] and Bhasin et al. [8] reported that MDS was the primary cause of micro-megakaryocytes.

A distinct shift towards immature megakaryocytes was the most noticeable morphological characteristic in 40% of ITP cases. Similar findings were also reported by Muhury M et al. [7]. Houwerzijl et al. [13] reported that this was due to the apoptotic and para-apoptotic forms of programmed cell death (PCD) in adult megakaryocytes. They claim that improper PCD of adult megakaryocytes may impair platelet formation and cause PCD (para-apoptosis), which mimics apoptosis and causes thrombocytopenia in ITP. Megakaryocytes still forming often resemble micro-megakaryocytes, which might deceive patients into believing they have ITP when Myelodysplastic Syndrome manifests as isolated thrombocytopenia [14]. The loss of megakaryocytes and aberrant maturation is caused by the antiplatelet autoantibodies to glycoprotein IIb/IIIa and Ib/IX seen in ITP, which prevent platelet formation and release [7].

Similarly to Bhasin et al. [8], Muhury et al. [7], and Choudhary et al. [10], emperipolesis was the prominent feature in all 100% of cases with ITP. Studies proposed megakaryocytes as a component of the marrow-blood barrier. They hypothesized that when there is an increased demand for cell delivery from the marrow into circulation, few cells choose the transmegakaryocytic route, resulting in emperipolesis [15]. They also linked emperipolesis to blood loss and suggested the patient be checked for any signs of bleeding.

In our study, cytoplasmic vacuolization was seen in 28.57% of cases of ITP, which was similar to the study done by Muhury et al. [7] and Choudhary PK et al. [10].

Houwerzij et al. [13] suggest that cytoplasmic vacuolation in megakaryocytes ultrastructurally reflects mitochondrial swelling, resulting from a higher turnover rate of megakaryocytes. Cytoplasmic vacuolization can also indicate two degenerative processes: apoptosis and para-apoptosis. Muhury M et al. [7] suggest that autophagy could cause this. It can be used to trap and break down specific pathogens or maintain cell metabolism in situations with an increase in metabolic demand and a nutritional deficit due to increased megakaryocytopoiesis. All of the above-described traits suggested dysmegakaryocytopoiesis, dramatically reducing platelet formation.

Limitations

Being a tertiary medical institution situated in the state capital, it serves the surrounding typical population, primarily urban, suburban, and outlying. A significant portion of the peripheral area needs to be screened. The results could have been different if the study had a greater sample size because the current study had a relatively smaller one. Further morphological and quantitative modifications from our research must be confirmed with a sizable study group, and patient follow-ups should not be considered conclusive. Finally, the emergence of the COVID-19 pandemic created challenges since it decreased patient intake throughout the study period.

## Conclusions

As a result of this investigation, it is clear that megakaryocytes in bone marrow aspiration of ITP patients experience considerable morphological changes. Due to the very common occurrence of dysplastic megakaryocytes in non-MDS hematological disorders, the threshold for dysplasia in megakaryocytes should be raised from the recommended 10%. However, further comparative study including cases of MDS has to be done to understand the significance of the occurrence of the dysplastic megakaryocytes in non-MDS-related thrombocytopenia. This study emphasizes the importance of morphological changes in the era of molecular genetic testing. So, the morphological study still holds great significance in places where other ancillary tests are not available. More research is required to compare and corroborate these findings. Future methods for computer-assisted diagnosis of certain diseases may benefit from such modifications. 
